# Digital Health Needs of Women With Postpartum Depression: Focus Group Study

**DOI:** 10.2196/18934

**Published:** 2021-01-06

**Authors:** Madison E Lackie, Julia S Parrilla, Brynn M Lavery, Andrea L Kennedy, Deirdre Ryan, Barbara Shulman, Lori A Brotto

**Affiliations:** 1 Women's Health Research Institute BC Women's Hospital + Health Centre Vancouver, BC Canada; 2 Department of Obstetrics & Gynaecology Faculty of Medicine University of British Columbia Vancouver, BC Canada; 3 School of Population and Public Health Faculty of Medicine University of British Columbia Vancouver, BC Canada; 4 Reproductive Mental Health Program BC Women's Hospital + Health Centre Vancouver, BC Canada; 5 Department of Psychiatry Faculty of Medicine University of British Columbia Vancouver, BC Canada

**Keywords:** postpartum depression, perinatal mental health, eHealth, women’s health, reproductive health, maternal health, qualitative research, focus groups, user-centered design, knowledge translation, self-management

## Abstract

**Background:**

Although approximately 10% of new mothers in Canada develop postpartum depression (PPD), they face many barriers when accessing care. eHealth offers a unique opportunity to provide psychosocial skills and support to new mothers; however, patient populations are not consistently engaged in eHealth development processes. Thus, the diversity of women’s backgrounds and needs are often not reflected in existing tools.

**Objective:**

This study aims to engage women from a variety of backgrounds and locations around British Columbia (BC) who have previously experienced PPD to determine the unmet psychoeducational needs of women with PPD and how a web-enabled platform used to deliver psychosocial skills and education to assist in the management of PPD could fulfill those needs.

**Methods:**

Focus groups were conducted in 7 cities across BC with a total of 31 women (mean age 34.5 years, SD 4.9), with each group ranging from 2-7 participants. Focus groups were cofacilitated by the study coordinator and a local service provider in each community using a semistructured guide to discuss participants’ needs, ideas, and opinions as they relate to the use of technology in PPD management. Transcripts were approached inductively using thematic analysis to identify themes and qualitative description to frame what was observed in the data.

**Results:**

A total of 5 themes were identified: bridging gaps to meet needs; providing validation to combat stigma; nurturing capacity to cope, manage, and/or reach wellness; empowering people to take ownership over their mental health; and offering customization to ensure relevance. Each theme identified a need (eg, combatting stigma) and a way to address that need using a web-enabled intervention (eg, providing validation). At the intersection of these themes was the overarching value of promoting agency for women experiencing PPD.

**Conclusions:**

Ultimately, new mothers require accessible mental health care that promotes their agency in mental health care decision making. Our participants believed that a web-enabled intervention could help meet this need. These data will be used to guide the design of such an intervention, with the eventual implementation of this resource as a first-line management option for PPD.

## Introduction

### Postpartum Depression

Postpartum depression (PPD) is a leading cause of maternal morbidity and mortality in high-income countries [1] and indiscriminately affects new mothers around the world. Defined in the 5th edition of the *Diagnostic and Statistical Manual of Mental Disorders* as a depressive episode with peripartum onset, including onset during pregnancy and for up to 4 weeks postpartum [2], PPD is a clinical illness that often requires professional medical and/or psychological intervention. However, research shows that the point prevalence of depressive symptoms appears to peak between 2 and 7 months postpartum [3]. Due to this discrepancy, it is likely that many cases are missed.

In addition to the symptoms of depression, many women also experience comorbid symptoms, including those of generalized anxiety disorder, panic disorder, social phobia, and posttraumatic stress disorder [4]. In Canada, 10% of new mothers experience symptoms consistent with PPD, and an additional 8% experience PPD and anxiety symptoms together [5]. When combined, this figure is approximately equal to the worldwide prevalence rate of PPD symptoms, which has been calculated to be 17.7% [6]. As such, PPD affects a substantial number of Canadian women each year and, if left unaddressed, can have significant short- and long-term negative impacts on the mother, child, and family.

A recent systematic review assessing 122 studies found negative impacts of PPD on mothers’ physical and mental health, infants’ physical health and development, mother-infant bonding, and familial factors, including finances and relationships with partners [7]. Outside of the immediate family structure, PPD can continue to have severe consequences. An economic analysis of perinatal mental illnesses in the United Kingdom found that the economic burden of these illnesses was UK £6.6 billion (US $8.9 billion) for live births in 2013, based on productivity losses, health and social care use, and resultant impairments and needs of the child [8]. When broken down to costs per woman by condition, perinatal depression was determined to cost UK £75,728 (US $101,748) per woman with the condition in the same year, with UK £10,237 (US $13,753) of the cost paid by the public sector [8]. On the basis of our similar sociodemographic makeup and socialized health care system, it is likely that Canadian figures align greatly with this estimate. As such, ensuring that women have access to effective, acceptable, affordable, and usable treatment options is a matter of societal and economic importance, in addition to advancing the health and wellness of individuals and families.

#### Barriers to Accessing Care

There is a wealth of available management options for PPD, with a strong evidence base supporting the use of psychological approaches, including cognitive behavioral therapy (CBT) and interpersonal psychotherapy; psychosocial options, including peer support and nondirective counseling; and pharmacological treatments, specifically antidepressants [9]. Despite these options, a large proportion of women experiencing PPD do not seek or engage with these options, even when referred [10-12]. The literature has consistently shown that help-seeking behaviors in these women are impacted by a wide range of barriers, including social, instrumental, and structural barriers [13-15].

The main social barrier, which can have wide-ranging effects on all levels of help seeking, is stigma. In many cases, societal and cultural stigma lead to feelings of shame and guilt and feed into some common fears for women experiencing PPD, including child apprehension [13-15]. These feelings can then compromise disclosure. On a more personal level, a lack of social support from family and friends can also create a barrier to accessing care and contribute to instrumental barriers that exist, such as a lack of childcare to attend appointments [13-15].

Financial constraints are another major instrumental factor in help seeking [13-15]. A recent study in British Columbia (BC) found a significant association between socioeconomic status and access to health services, indicating a gap in care for women of low socioeconomic status who may already be underserved [16]. This is especially true for mental health services, as Canadian health care systems generally only cover psychiatric and community-based mental health services. The concentration of care options in urban centers can also be detrimental, as transportation and other indirect costs of accessing care are often identified as barriers to accessing care [13-15].

Finally, there are structural factors that impact access, with a lack of knowledge and information identified as a key element, both in patients and care providers [13-15]. This can lead to women remaining undiagnosed for longer than is necessary, and once these women are identified and directed to resources, they may be met with provider barriers, such as a lack of culturally safe care, and wait times that continue to impede their help seeking [13-15].

Throughout each sphere of life, women face barriers and gaps in accessing in-person care that contribute to and exacerbate their experiences of PPD. As such, there is a need to create and implement mental health management resources and interventions that address the inescapable stigma present in different cultural and social settings and that are effective, affordable, and accessible.

### Uses of eHealth in Mental Health

The field of eHealth, involving the translation of psychological and other health interventions via telephone, apps, or web-based platforms, has been instrumental in the movement toward making health care accessible and personalized. By making information, education, and management resources readily available, eHealth allows individuals to “become partners in their own health” [17], empowering them to make decisions alongside their care providers.

eHealth interventions have been created and assessed for a wide range of common mental health conditions and are considered a cost-effective and accessible way to deliver care [18]. Several such interventions have been created using computerized CBT as the foundation of the provided therapeutic modules and have demonstrated efficacy in treating common mental health disorders, including depression and anxiety [18-21].

In addition, in recent years, there has been the introduction of multiple web-based interventions specifically for the treatment of perinatal mental health conditions. Two recent systematic reviews found that these interventions are moderately effective (effect size Hedges *g*=0.60) at improving maternal mood; however, both indicated a need for more research in this area to provide additional evidence and address existing limitations [22,23].

In the literature, accounts of these existing interventions for perinatal mental health are found around the world, covering a range of therapy types and modes of delivery. A group in the United Kingdom created Netmums, an intervention that provides guided behavioral activation treatment [24,25]. An intervention created jointly by groups in the United States and Australia provides a CBT-based program with personal coach assistance, called MomMoodBooster or MumMoodBooster, depending on the location [26-28]. A Swedish team created a CBT program based on an existing, established internet CBT program used in Sweden [29]. A Canadian group created a therapist-assisted CBT program [30]. In randomized controlled trials, each of these interventions demonstrated significant reductions in depressive symptoms following treatment compared with control groups [24,25,28-30].

However, only some of these interventions have published information about patient engagement during the design and development of these platforms, and there are still gaps in creating a truly user-centered tool. The Canadian therapist-assisted CBT program assessed qualitative feedback from clients who used the researcher-designed program during a randomized controlled trial and identified areas of improvement for the future [31]. As such, the resulting interface seems to be entirely designed and developed by the research team, with little involvement from end users. Meanwhile, the program content for Netmums was based on a previous qualitative inquiry using semistructured interviews, which assessed women’s needs and preferences for perinatal-specific CBT [32]. However, this work did not focus on an eHealth solution; therefore, there may still be unidentified gaps that come from employing technology for therapeutic purposes. Conversely, the MomMoodBooster program received significant qualitative feedback through the use of focus groups and usability testing to identify user needs specific to a web-based program [26]. However, these focus groups involved the presentation of preidentified content and user interface features, which were then adapted. As such, there may be missing features that could have been identified through a priori discussions.

As outlined in the established standards for evaluating eHealth interventions, a crucial time for evaluation is during the conceptualization and design phase [33]. However, research has shown that many eHealth interventions are only evaluated at the end of the study period, once the intervention has been fully developed and tested [34]. This study aims to inform the design and development of a proposed web-enabled intervention by first identifying the gaps in care and assessing the needs, values, and preferences of women experiencing PPD. As such, it employed the principles of integrated knowledge translation, which asserts that “involving knowledge users as equal partners alongside researchers will lead to research that is more relevant to, and more likely to be useful to, the knowledge users” [35]. Similarly, this aligns with the ideals of user-centered design, which is an iterative process of web design that involves end users throughout the development lifecycle [36]. A user-centered design establishes the need to know who end users are and to involve them early and often, to design tools that are relevant and useful [37]. A more significant inclusion of participants during the formative phases of content design and development would likely be beneficial in creating more applicable, effective interventions.

### Objectives

This was the first stage of a multiphase study aiming to develop a web-enabled intervention that delivers psychosocial skills and education to women experiencing PPD. This preliminary phase had the following objectives: (1) to determine the unmet psychoeducational needs of non-Indigenous, nonmigrant women with PPD and (2) to explore how a web-enabled intervention could help to meet those needs, including what specific features and content this intervention must have for women to use it. Future phases of this study aim to collect similar data from both Indigenous and immigrant women.

## Methods

### Overview

As the first stage of a multiphase project, this study used focus groups to explore and understand the needs, preferences, and experiences of non-Indigenous, nonmigrant Canadian women who have experienced PPD to inform the development of a web-enabled intervention to support PPD management. This study will inform future studies assessing the needs of Indigenous women and immigrant women in BC to ensure that the intervention is accessible and acceptable to Canada’s diverse populations.

### Study Population

To ensure that a diverse set of experiences and needs were included, a purposive sample of participants was recruited from 7 communities across BC (Table 1), varying in location, size, population demographics, and access to resources. These locations included Vancouver, Burnaby, Surrey, Kelowna, Prince George, Comox, and Victoria. In addition, women who lived outside of these specific communities, but who were interested in participating and lived in the general vicinity, were invited to attend focus groups in person or via Skype.

**Table 1:** Number of participants in each group location.

Location	Participants, n	Age (years), mean (SD)	Number of children, mean (SD)
Surrey	7	34.7 (5.6)	1.6 (0.5)
Vancouver	6	36.0 (3.5)	1.5 (0.5)
Burnaby	5	32.2 (4.4)	1.4 (0.5)
Prince George	2	34.0 (7.1)	2.0 (1.4)
Kelowna	5	31.0 (4.3)	2.2 (0.4)
Victoria	4	36.5 (4.9)	1.5 (0.6)
Comox	2	40.0 (5.7)	2.0 (1.4)
All locations	31	34.5 (4.9)	1.7 (0.6)

#### Eligibility

Eligibility criteria were set to ensure that focus group participants were able to provide insightful reflections on the helpfulness of mental health management strategies for PPD. To meet the inclusion criteria, participants were required to be 18 years or older; assigned female at birth and identify as a woman; identify as a non-Indigenous Canadian; be able to read, write, and speak conversational English; and have experienced PPD within the last 5 years but no longer meet the diagnostic criteria. All inclusion criteria were self-reported by participants. Before enrollment, participants were screened for current depressive symptomatology over the phone using the Edinburgh Postnatal Depression Scale (<12), which is the screening tool currently recommended by Perinatal Services BC [38].

#### Recruitment

Ethics approval was obtained from the University of British Columbia and BC Children’s and Women’s Hospital Research Ethics Board. Ethical considerations included providing all participants with a list of mental health resources available on the web and across the province of BC following the phone screen process and following each focus group because of the potential for triggering distress. In addition, a protocol was developed for participants who endorsed thoughts of self-harm, which ensured that they had access to mental health supports. Purposeful sampling was used to identify participants, with enrolled participants encouraged to aid in snowball recruitment through their own social networks. Study advertisements were promoted through social media and local service providers, with the majority of participants responding to ads on local Facebook groups, and through the Pacific Postpartum Support Society—a community partner in the Lower Mainland of BC, which encompasses the largest urban population in the province.

### Procedures

Three distinct phases of participation were created, including completion of a demographic questionnaire, attendance at one focus group session, and an optional opportunity to take part in member checking through reviewing participants’ own transcripts.

#### Demographic Questionnaire

A demographic questionnaire was completed by all enrolled participants; the questionnaire collected information relating to participants’ personal life experiences, including data on socioeconomic status, intimate and family relationships, and medical history. Participants were offered the option to complete the questionnaire at home via an emailed unique survey link or mailed paper copy or at their focus group session on an iPad provided by the researchers. Study data were collected and managed using Research Electronic Data Capture tools hosted at BC Children’s Hospital [39]. Descriptive statistics were analyzed using Statistical Package for the Social Sciences (version 26.0; IBM) [40].

#### Focus Group Interviews

As described earlier, focus groups were conducted in 7 communities across BC, with non-Indigenous, nonmigrant Canadian women. It is well established in the focus group methodology literature that sociocultural homogeneity within the group can be an important factor in creating a more comfortable and open sharing environment [41,42]. As such, Indigenous and immigrant focus groups were held separately in the second phase of this research.

Focus groups were held in local public venues, such as libraries and community centers. Skype participation was available for participants who were unable to attend a group in person because of unforeseen circumstances but who still wished to attend remotely. In these cases, Skype participants attended the same session as in-person participants but were displayed on a monitor for the in-person participants to see, with a camera setup so that the remote participant could also see the group. All study procedures for Skype participants were identical to those of in-person participants, via remote participation. Two participants chose to attend focus groups via Skype, and these participants were involved in the 2 rural focus group sessions in Prince George and Comox. These focus groups ranged from 1 to 2 hours in length, depending on the number of participants and time it took to thoroughly discuss emergent topics.

In addition to the study coordinator acting as a facilitator, each focus group employed an experienced cofacilitator from the local community to create a more accessible and acceptable environment for the participants. Cofacilitators were mainly service or care providers and included a public health nurse, a PhD student in clinical psychology, a family support worker for a pregnancy outreach program, the director of the Pacific Postpartum Support Society, and the study principal investigator who is a registered psychologist. These collaborators were recruited through general internet searches for local services for new parents and those who may be experiencing PPD. To ensure that any potential power dynamics between participants and providers were acknowledged, cofacilitators briefly discussed at the beginning of each focus group their role in the group and the community and encouraged participants to feel open to share their experiences, both good and bad, without fear of repercussion. In addition, included in the ground rules set out in the focus group interview guide to ensure participant safety and comfort, the facilitators would state the following: “There are no ‘right’ or ‘wrong’ answers, just different opinions. We ask that you respect each other’s opinions and experiences, and say what is true for you without fear of judgement” (Multimedia Appendix 1).

The research team created a semistructured interview guide, which was not piloted before collecting study data. The interview guide posed a series of questions that transitioned from an open discussion of participants’ experiences with PPD and available treatment options to more specific questions regarding their ideas for the design of the proposed web-enabled resource, including interactive features, peer support options, and the inclusion of partners. Each group concluded with a discussion of what participants regarded to be the most important factor in their personal recovery and what would be necessary for the research team to consider when designing the eventual platform. At least one focus group was run in each community, with no further groups run when saturation was reached, that is, no new ideas or information were observed [43].

In all groups except for one, because of time constraints, a discussion of an existing web-based CBT program called BounceBack was included, in addition to the regular interview guide, to inform the design and delivery of a potential CBT component in the proposed web-enabled intervention. BounceBack is a physician-recommended website available across Canada [44]. It has a significant evidence base to support its efficacy in managing a range of mild to moderate mental health disorders, including anxiety, depression, and eating disorders [45-47]. The group discussion of BounceBack was used to inform the design and delivery of CBT through the proposed web-enabled intervention and entailed an interactive walk-through of the website and a discussion of its strengths, weaknesses, and relevance to PPD.

To increase accessibility, women were encouraged to bring their young children if necessary, provided a catered meal during the group, and offered a $50 honorarium and parking reimbursement. Each session was audio recorded and transcribed verbatim by a contracted company, with additional context provided by field notes taken by a research assistant during each group. Before transcription, each employee at the transcription company was required to sign a confidentiality agreement, and participants were informed of this process to ensure that their informed consent was provided.

#### Member Checking

Participants were provided the optional opportunity to review their transcripts before analysis to honor participants’ ownership of their experiences, how they were represented, and how they were interpreted to inform the resultant web-enabled intervention. If participants chose to take part in this step, they were sent the anonymized transcript from their focus group and informed of their study ID so that they could identify their individual comments. Participants were then asked to provide feedback on the accuracy of their transcript, any information that they would like redacted or clarified, and any unidentified portions of the transcript that were attributed to them, for example, if the transcriber was unable to determine who was speaking. Member checking substantiates the trustworthiness and transferability of results as participants confirm their dependability and credibility [48]. Of the 31 participants, 7 participated in member checking. Moreover, 4 of the 7 participants provided minor feedback related to clarifying words that were unintelligible or changing background information to be less identifiable. None of the changes made impacted the resultant analysis.

### Qualitative Analysis

Anonymized, verbatim focus group transcripts were analyzed using the qualitative analysis software NVivo 12 (QSR International), which allowed for streamlined data management and synthesis across multiple research staff [49]. The 3 researchers who performed the analysis—all of whom were White, cis-gendered, nulliparous women—approached the data guided by a social constructivist framework, thus exploring and acknowledging how the data were shaped by who was in the room, opportunities to speak, and perceptions of safety in sharing during the focus groups [50].

The team used a qualitative descriptive approach to data analysis, identifying themes inductively and thematically. Qualitative description (QD) is often used in health research to inform the development of interventions or policies that can improve health outcomes for various populations [51]. On the basis of exploring “the who, what, and where of events and experiences” [52], QD provides a straight description based on participants’ responses, making use of participants’ own language to support the themes that emerge [52-54]. Its usefulness in informing interventions and its ability to assist in translating findings directly and rapidly to improving care makes QD a well-suited approach for the development of a web-enabled intervention [51]. Consistent with the literature for QD, inductive thematic analysis was used to explore and explain the focus group data [53]. Thematic analysis is described as a method of “identifying, analyzing, and reporting patterns” that exist within a qualitative data set [55].

In assessing the theoretical strength of qualitative research, there are a number of reputable frameworks, including the criteria developed by Lincoln and Guba [56] to assess trustworthiness, which include credibility, transferability, dependability, and confirmability. In this study, the development of the names, definitions, and relationships of codes, categories, and themes based on the approaches of Miles et al [57] were tracked via reflexive memos with illustrative quotes. In addition, each transcript was coded by a single investigator, and the resultant coding was then reviewed by a second investigator, with disagreements in coding discussed by the whole research team to arrive at the accepted analysis. This strengthened the dependability and trustworthiness of the results, as these practices facilitated the 3 investigators’ intercoder reliability [58,59]; triangulation [60]; and arrival at a shared vision with participants, as confirmed via member checking [48]. When reporting participants’ responses, their age, focus group location, and number of children were provided to contextualize the information and lent itself to the credibility, transferability, and dependability of the findings [61]. The creation and use of high-quality data is a significant step toward informing accessible, acceptable, and relevant care for women experiencing PPD across Canada.

## Results

### Demographics

A total of 31 women participated in the 7 focus group sessions (Table 2). The mean age was 34 years (range 24-44), with a mean of 2 children (range 1-3). This sample of women was predominantly White, highly educated, and married.

**Table 2:** Sociodemographic characteristics of focus group attendees (N=31).

Variable^a^		Value
Age (years), mean (SD)		34.5 (4.95)
**Sexual orientation, n (%)**		
	Heterosexual	29 (94)
	Bisexual	2 (6)
**Ethnicity, n (%)**		
	Chinese	2 (6)
	Hispanic or Latin American	1 (3)
	South Asian (East Indian, Pakistani, Sri Lankan, etc)	1 (3)
	White European	25 (81)
	Biracial	2 (6)
**Education, n (%)**		
	Graduated high school or earned GED^b^	2 (6)
	Attended some college or university	4 (13)
	Graduated 2-year college or university	8 (26)
	Graduated 4-year college or university	9 (29)
	Postgraduate degree	8 (26)
**Employment, n (%)**		
	Full time	10 (32)
	Part time	5 (16)
	On maternity leave	5 (16)
	Stay-at-home caregiver	7 (23)
	Student	2 (6)
	Unemployed	2 (6)
**Annual household income, n (%)**		
	Less than Can $20,000 (US $15,634)	2 (6)
	Between Can $20,000 and $39,999 (US $15,634 and $31,266)	2 (6)
	Between Can $40,000 and $59,999 (US $31,267 and $46,900)	7 (23)
	Between Can $60,000 and $79,999 (US $46,901 and $62,534)	3 (10)
	Between Can $80,000 and $99,999 (US $62,353 and $78,167)	4 (13)
	Between Can $100,000 and $119,999 (US $78,168 and $92,801)	2 (6)
	Between Can $120,000 and $139,999 (US $92,802 and $109,434)	2 (6)
	Between Can $140,000 and $159,999 (US $109,435 and $125,068)	2 (6)
	More than Can $160,000 (US $125,069)	5 (16)
	Prefer not to answer	2 (6)
**Relationship status, n (%)**		
	Never married	1 (3)
	Dating	1 (3)
	In a relationship	1 (3)
	Married	25 (81)
	Common law	1 (3)
	Separated	2 (6)
Number of children, mean (SD)		1.7 (0.65)
Number of PPD^c^ diagnoses, mean (SD)		1.1 (0.30)

^a^Percentages may not equal 100 because of rounding.

^b^GED: general education development.

^c^PPD: postpartum depression.

### Themes

Five major themes emerged: bridging gaps to meet needs; providing validation to combat stigma; nurturing capacity to cope, manage, and/or reach wellness; empowering people to take ownership over their mental health; and offering customization to ensure relevance. Each of these themes identifies a need (eg, combatting stigma) and a way to address that need (eg, providing validation).

#### Bridging Gaps to Meet Needs

Participants identified gaps that exist in knowledge about PPD and care options for PPD. They explained how this contributes to unmet needs, such as being unaware of the manifestations of PPD and finding care to be inaccessible. Opportunities for a web-enabled intervention to assist in bridging these gaps in the interests of meeting their needs were discussed.

The gaps described by participants ranged from access to care (ie, eligibility), care provision (ie, availability and acceptability), and awareness and understanding of postpartum mental health concerns. Multiple participants identified that the current definition of *postpartum* compromises responsiveness to their needs and contributes to their needs remaining unmet, as illustrated by the following participants:

...because one thing I have to say is the postpartum period is not a year. And that, that bothers me because I was in a race the first time for this to be done with. [...] I struggled for four years after my second.

SUP21, 36, Vancouver, 2 children

...so, I only saw (doctor) the one time. [...] Then when I called back and my son was already a year old, they said, “Well, it’s too late.” Like, I guess they only offer help for up to a year.

SUP7, 39, Surrey, 2 children

They explained how a web-enabled intervention could bridge these gaps:

I guess that seems like it might be a nice advantage of an app. Like, you wouldn’t need to be one-year. Under one-year postpartum. You can just access it any time. And benefit from it.

SUP28, 31, Burnaby, 1 child, 2 additional children as surrogate

Even when eligible for care—for example, less than 1 year postpartum—many participants discussed the barriers they faced in accessing available options, such as the timing of appointments for care and the impact of long wait times to see specialists:

...I got referred to the group so often. It’s like, “Yes, you need help. This is the help option,” and I couldn’t access that option. [...] I have friends that volunteered to call in sick so they could look after my son [tearful voice] so I could go, you know? And it’s just that option wasn’t there.

SUP25, 42, Victoria, 2 children

This was in November, and they weren’t able to give me an appointment until March. So unfortunately, I wasn’t diagnosed until March, like, officially, and that kind of, like, does break my heart and breaks my husband’s heart.

SUP12, 37, Burnaby, 1 child

A web-enabled intervention was supported as a way to provide care to women who face these barriers, either as a first-line intervention, or as a supplement to in-person care:

[...] in my situation here, because resources are scarce [...] especially in northern, rural B.C., it’s hard to access things, so whether that be a counsellor or sometimes even a good internet connection, right? So, I would need a resource to be as self-directed as possible.

SUP13, 29, Prince George, 3 children

[It] would be great for supplementing and helping a woman before they actually get to see a therapist or start on their medication or something and then would support them through that as well.

SUP11, 32, Surrey, 2 children

A pervasive topic across the focus groups was a lack of awareness and understanding regarding PPD. Awareness was defined as knowing of the existence of PPD as a mental health concern, whereas understanding went deeper into knowing *about* PPD, including symptoms and potential treatment options. Participants expressed how a lack of knowledge in their friends and family regarding the existence and presentation of PPD could contribute to unmet needs. A web-enabled intervention was endorsed as a way to engage and educate these support people to better assist women by providing information about PPD, ways to help, and how to access care:

Well, I think it’s important for [partners] to be involved. I don’t think that, they don’t understand either. Like, they don’t, I know mine wasn’t. Like, he had no idea.

SUP26, 29, Prince George, 1 child

Or even signs, too, because I think, a lot of times, they notice that things are going on but don’t necessarily know what to do with that information or what it’s really reflecting, especially if they don’t have any [experience], they’ve never even been exposed to information on postpartum depression.

SUP32, 30, Kelowna, 2 children

Although this investigation was focused on the role of a web-enabled intervention in mental health management, most participants expressed the value of its use as a preventive and promotive tool to feel better equipped in what to expect from the postpartum period:

I almost wish they made more of a case of it during, like, your pregnancy, you know? Like, “You should anticipate this. This could totally happen to you.”

SUP28, 31, Burnaby, 1 child, 2 additional children as surrogate

I don’t know if I would have been able to really look at anything while I was in it, but I think beforehand, like if it was available to people that are pregnant, then that might help [...] if I had already been on that app beforehand and, you know, knew what it was all about, then I would have been more open to that.

SUP1, 32, Surrey, 1 child

Unmet needs, such as a lack of knowledge regarding PPD, were thought to persist because of gaps that exist and are perpetuated by stigma, and participants have underscored how the normalization and validation of postpartum mental health is an effective way to combat stigma:

[...] normalization of the experience helps because most of us are sobbing in our homes, you know, thinking that this sucks. And we have nobody to tell, and there’s guilt and shame and all this stuff that comes with it. And it’s actually really common [laughter], um, but [...] it’s supposed to be positive, right?

SUP32, 30, Kelowna, 2 children

#### Providing Validation to Combat Stigma

Participants spoke about their experiences with stigma, especially as it relates to notions of being a good mother. They described how it can become internalized and its effects on their mental health. For example, 1 woman expressed:

Like, for me, for example because I was feeling so, um, like, um, low, and like so hard on myself, feeling inadequate. You know, like, maybe I should be suffering, so when my husband said, “You should take care of yourself,” it’s like, “No. Like, I deserve to not feel good.”

SUP41, 31, Victoria, 1 child

Validation of their feelings and experiences was key in addressing this internalized stigma, both through education and normalization of postpartum mental health concerns before they occur and through the sharing of stories from other women who have had PPD to fight feelings of isolation:

[...] in the support group, I never felt that. Like, everybody was saying their truth. It was like, “Oh, she is experiencing that. Wow. Okay. I am okay.” There was no repercussion. It was just a safe place to, to feel okay.

SUP8, 43, Surrey, 2 children

Examples of how a web-enabled resource can integrate these features were provided, with 1 participant describing the inclusion of peer and professional encouragement:

Yeah, I think just some way of encouraging women whether it be through success stories or a connection with a counsellor, whatever it is, through the app that, um, would just encourage them not to give up and encourage them that it will get better.

SUP30, 36, Comox, 1 child

Another participant described what she found useful in the resources she used and how they could be adapted:

And seeing that stuff, I don’t know if it was for you guys too, but, like, knowing that it’s not, you’re not crazy. It was just relief to know. I mean, when you read that, you’re like, “Oh my gosh. This is totally what I’m going through.” And it was so relieving, so having stuff on the app like that would be [good].

SUP23, 39, Victoria, 1 child

In addition to appreciating validation, participants described how they would want to reciprocate that validation by mobilizing their experiences and knowledge in the interest of demystifying and normalizing PPD, raising public awareness and combatting stigma:

Every chance I get to talk about my experience, I do. Like, I posted [...] on Facebook, and I asked everyone to share it. [...] I want someone to feel better, so I think just talking about it is important. Yeah, so you don’t feel like a crazy person, right?

SUP26, 29, Prince George, 1 child

They identified that pervasive stigma toward PPD impacted attitudes expressed by family and friends, which was damaging to their mental health:

[...] so like, my mom would be like, “This is so weird. I have no idea what’s wrong with you.” Like, she’d actually say that to me. Yeah. She’s like, “I loved my babies instantly. Like, as soon as I saw them, I was, like, in love.” I’m like, “Oh God.”

SUP41, 31, Victoria, 1 child

The effects of stigma were woven throughout personal and social interactions and greatly impacted the mental health and help seeking of women experiencing PPD. However, through these experiences, they discovered the power of validation and normalization to combat these attitudes. They also described lessons for nurturing capacity in other women to assist their journey to wellness:

I think it would be helpful in terms of normalizing it. Because I think the more resources via app or in person or whichever, helps to normalize it. Yeah, the more information you have, and the more, and right at your fingertips because you can’t always leave the house. Right?

SUP30, 36, Comox, 3 children

#### Nurturing Capacity to Cope, Manage, and/or Reach Wellness

The need for tools to assist in management was clear when participants identified the challenges they faced in coping with and managing their mental health concerns and on their journey to wellness:

Yeah, I’d add, like CBT is great, but you have to have so many other things in place before it works. [...] I think a lot of people who don’t have their basic practical needs met, like, “I don’t actually have time to have a break because I have nobody to help me.”

SUP10, 30, Vancouver, 1 child

I knew that there was something really wrong, so I went, I think, once a week for support group and we did, my doctor tried to get me on meds, too, and I did not want to, so I just went to the support group. We learned meditation. We learned CBT, which was really helpful.

SUP8, 43, Surrey, 2 children

Also, I, like, had extreme panic attacks about taking the medication. I was so afraid [...] And so I worked with a therapist for probably four sessions before I even took one. [...] within a month, I was like, “Holy shit. I am like a complete, I love my child. I love my life. Wow, the sky is blue.” I was like I can’t believe I was so worried about taking medication that has completely changed my life and made me such a better mother.

SUP15, 36, Vancouver, 1 child

Many participants described how a web-enabled intervention could be used to nurture their capacity, thus providing the tools and education necessary to facilitate their ability to reach each of these levels of mental health management. Often, these tools were identified as being most helpful before mental health concerns arise, when they could be used as a preventive measure. However, once PPD occurs, many women emphasized the value of connecting them to peers with lived experience and providing evidence-based knowledge to deepen their understanding of PPD and the ability to determine their path to wellness:

I think there’s a lot of benefits to having both peers and professionals on it? Because you, you’re gonna trust a professional. They’ll tell you, “Drink 20 cups of water,” and you’re going to be downing the 18th one. As opposed to, like, a peer. A peer is a little bit more approachable, right? And you’re almost...you’re on the battlegrounds with them, so there’s a lot of validity in both.

SUP19, 36, Vancouver, 1 child

I was obsessed with the question, “Would I ever get better?” and I would ask everybody. [...] I would just plead with everybody, “Does this end?” So, to have success stories built into the app. Might be really helpful.

SUP10, 30, Vancouver, 1 child

[...] a community hub or a portal where, like, all that information is just. In one place. Because [...] I didn’t like reading about it. But the little bit I did read was from different sources, so if you just had everything in one place.

SUP12, 37, Burnaby, 1 child

They also discussed how gaining knowledge from peers and professionals can lead to personal insights and suggested ways of including opportunities for self-reflection to promote these insights within a web-enabled resource:

[...] mood tracking and stuff as just a way of, uh, paying attention to it, I think, was the point. Because a lot of the time when you’re depressed, you’re not...really thinking about the specifics of why you feel like you feel.

SUP10, 30 Vancouver, 1 child

Besides educating themselves, participants endorsed engaging family and friends to assist in nurturing their capacities to cope, manage, and reach wellness:

Yeah, I think anyone who might be involved, like someone, if they don’t have a partner or maybe if it’s their roommate or their mom or whoever it is that’s kind of closely tied to them. That could be that support partner.

SUP30, 36, Prince George, 3 children

Participants explained how having their experiences validated and their capacity nurtured empowered them to trust that they knew what their needs were and how best to meet them. However, they also discussed how having their capacity minimized and their needs dismissed by those they seek support from can leave them feeling disempowered and as though they have no control over their care:

[...] when I talked to the person [...] on the help line, um, [...] it was kind of strange, but I, because she almost sounded, made it sound like I was some kind of helpless victim of this thing, you know? But, which to me, didn’t sound like, [...] not very empowering [...] And I told her, “I am not a helpless victim. I know I can’t help the hormones and all of that, but there are things I can do.”

SUP43, 44, Prince George, 3 children

#### Empowering People to Take Ownership Over Their Mental Health

When feeling disempowered, participants often reflected on power dynamics within the health care system. Specifically, how their perceived lack of power was a detriment to their mental health and how a web-enabled intervention could promote agency in their pursuit of mental well-being:

I didn’t want to do medication, which is what I felt the psychiatrist just kind of pushed. She was very nice about it, but she kind of, I felt like she pushed it too hard.

SUP2, 29, Surrey, 2 children

[...] did I get help? I don’t think it was helpful. I think that the midwives thought it was helpful.

SUP9, 39, Surrey, 2 children

I find certain questions very intimidating because...what’s going to happen if I say, if I say I am having thoughts of hurting myself or someone else, it doesn’t mean I need to be slammed into a cage.

SUP9, 39, Surrey, 2 children

When describing how a web-enabled intervention could bridge these power dynamics, they discussed how it could supplement their relationships with care providers and ensure that they are able to make informed choices as partners in their own care:

[...] like, even if it was like, “Hey, if you’re in B.C., like, this is what you need to know in B.C.” Like, step by step by step, “Do this. Do this. Do this.”

SUP12, 37, Burnaby, 1 child

A common idea across focus groups centered on tracking your mental health using a web-enabled intervention to facilitate care:

Then yeah, if you are seeing someone [...] you can take out your app, and you can tell them, “Actually, I’ve noticed that over the last week, I’ve been more anxious. Let’s talk about that” or “I’ve noticed that when I send my son to school, I start feeling like this.”

SUP11, 32, Surrey, 2 children

In addition, they discussed the importance of their personal insights in determining the best pathway for them to achieve mental well-being. For example, identifying preferences in care options, such as self-care versus specialist care. Many identified self-care as a necessary way to make up for gaps in other care options:

[...] the piece that really turned the corner for me was [...] she drew a teapot, and she said [...] “You have all the things going into the tea kettle that are creating the pressure.” So, she listed all of the things that I had talked about, and she said, “You have no outlet to let the pressure out, so and now your brain is exploding, basically.” [...] And so when I feel that buildup of rage, because it usually expresses itself as rage, that’s what I think about like, “My teapot is going to explode. How do I relieve the pressure?” So, I either take the pressure off myself and leave things that are putting pressure on or I go for a walk or I do something else that’s going to help me to calm down. And I know what those things are, so I have my tools in my toolbelt, right?

SUP25, 42, Victoria, 2 children

Participants expressed how a web-enabled resource could support them in informing, understanding, and realizing their self-care practices:

I would like to see something that popped up, like a pop-up that said, “Self-care” [laughter]. Because this is a word I had no idea existed until postpartum.

SUP8, 43, Surrey, 2 children

Overall, participants considered how a web-enabled intervention could assist women by nurturing their capacity and empowering them to reach wellness on their own terms. They also identified how ensuring relevance is integral to these goals.

#### Offering Customization to Ensure Relevance

Participants suggested how a customizable interface through which people curate their journey toward mental well-being could offer relevance and make management more effective. Across themes, participants spoke about how current strategies and supports do not reflect their pregnancy and postpartum experiences. Consequently, the need to ensure relevance was highlighted throughout their discussions. Specifically, participants explained how a web-enabled intervention could offer customizable features to tailor resources to meet their needs:

I think that’s a really important point for an app because it can’t be one thing for all people. It needs to be, um, personalized to your, you know, what would help you the most.

SUP24, 34, Victoria, 2 children

On the basis of these customizations, women would then be able to access resources and support on their own terms:

[...] just, like, a tiny bite, just a tiny bite because that’s all I could have taken. And then maybe once I’ve taken that bite, there are, like, bigger bites going down. [...] so, like, little, tiny bread crumbs to be like, “Try this” or “Try that” and then, “If you try it and you like it, well here’s a something a little bit different but the same. It might help you differently, better.”

SUP31, 32, Kelowna, 2 children

Yes, I think maybe different levels, right? Different levels because sometimes you just need a mom that understands. And then sometimes you need to go to a suicide hotline.

SUP1, 32, Surrey, 1 child

They also reflected on their preferences for content delivery strategies and recommended interactive features. These contributions were often informed by their responsibilities and demands (eg, time constraints) as parents and what would be considered accessible and acceptable:

So even if it takes 20 pages, but 20 pages of smaller information because then it’s easier to be like, “Okay. I was on page 5. I’ll continue it later,” rather than getting lost on a whole book [...] but something you can just quickly scroll through while you are breastfeeding, feeding a child, or anything, right?

SUP2, 29, Surrey, 2 children

You almost have to, like, you almost need to BuzzFeed it [laughter], you know? [...] I’d love to see, like, you know, “Three self-care tips you can do right now.”

SUP12, 37, Burnaby, 1 child

I think, like, if you don’t want to sit there and read for. Then you can watch the video. You can, like, pick and choose out what you want or what you think relates to you.

SUP26, 29, Prince George, 1 child

**Figure 1:**
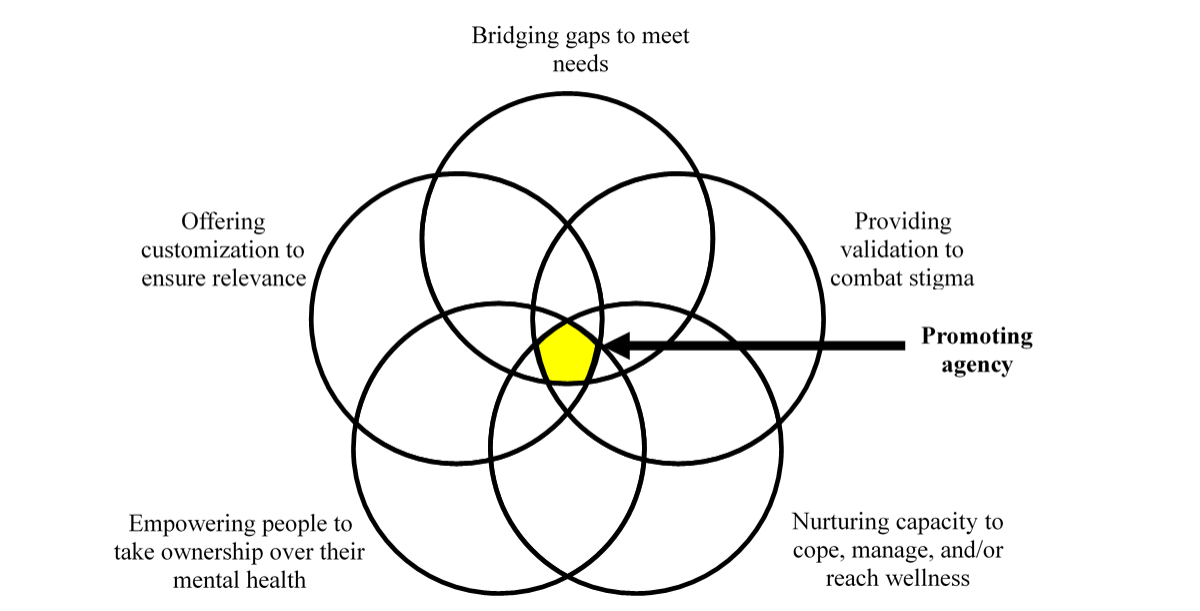
Intersection of the 5 themes leading to the overarching goal of promoting agency.

Ultimately, participants identified gaps and expressed how a web-enabled intervention could fill their needs for validation, increased capacity, and empowerment by offering relevant content. At the intersection of these 5 themes exists the overarching goal of promoting agency in women experiencing PPD so that they can determine and pursue the most effective and acceptable path to personal mental health and wellness (Figure 1).

### Integration of Research Findings to Define Next Steps

To actualize the preferences of participants, it was necessary to explore specific recommendations for how their needs could be met through the use of a web-enabled intervention. Explicit examples of this were provided across the focus groups, with participants describing key features related to the emergent themes (Textbox 1). Moving forward into web development, the research team will integrate these features and content areas to ensure a user-centered, patient-oriented tool is created.

**Overview of how the emergent themes relate to specific features and content details.**

Bridging gaps to meet needs

- Pathway to local services
- Self-directed therapies (cognitive behavioral therapy, mindfulness, etc)
- Availability throughout maternal trajectory

Providing validation to combat stigma

- Chat forum (with moderators)
- Peer buddy system
- Blog posts to share stories

Nurturing capacity to cope, manage, and/or reach wellness

- Resource hub
- Evidence-based information
- Resources for support people

Empowering people to take ownership over their mental health

- Daily prompts/check-ins
- Mental health tracking
- Self-care information
- Resources for holistic care

Offering customization to ensure relevance

- Profile/baseline assessment
- Options for opting in/out of features
- Content delivery for different learning styles (ie, written, videos, audio, etc)

## Discussion

### Making Connections

This study aims to determine the unmet needs that persist for women experiencing PPD and to explore how a web-enabled intervention for PPD could assist in meeting those needs. These findings clearly demonstrate that there is a range of unmet needs, including a need for education, validation, empowerment, and accessible care.

This reflects the current knowledge base regarding women’s perceived barriers to accessing care and their unmet needs in PPD. As described throughout our themes, these gaps exist on multiple fronts and at multiple levels of mental health management. Stigma is consistently described as a significant barrier to help-seeking behavior [13-15]. As seen in this study, and as supported by previous literature [13], cultural stigma related to pregnancy and postpartum experiences can lead to guilt and shame in women. In addition, participants demonstrated how a lack of validation and normalization of postpartum mental health concerns exacerbates these feelings [62].

Woven throughout the themes is a lack of knowledge and understanding of postpartum mental health—another significant barrier to the pursuit of mental well-being [13-15]. This is directly related to stigma, validation, nurturing capacity, and empowerment, and much of what participants discussed in these themes involved feeling alone in their experiences, not having the information and tools to manage their mental health, and requiring education to become partners in their journey to mental well-being. A lack of knowledge regarding PPD in general and available resources and supports has been identified consistently in the literature, contributing to unmet needs [13-15].

Our themes clearly point to the importance of providing a resource that can be tailored to, and addresses, the diversity of needs and complexity of experiences faced by women experiencing PPD. In addition, ensuring that mental health resources are made accessible to women, regardless of factors such as location or income, is crucial.

Despite this sample of participants being predominantly well educated and affluent, the majority struggled to access care for PPD. However, financial constraints (which could be linked to income) and a lack of knowledge about PPD (which could be indirectly impacted by education level) are consistently highlighted as barriers to accessing care in the literature [13-15]. The financial aspect is especially important in Canada, where the universal health care system funds only psychiatric and community-based mental health services and pharmacological treatments [63]. This suggests that mental health concerns and care are prohibitive, even for those of affluent status. It also seems that social factors, such as stigma and social support, and structural factors, such as location, wait times, and health care provider knowledge, may play a strong role in facilitating access to care options for this population.

eHealth may provide critical access to information and services through its accessibility and affordability. In 2018, internet connectivity was available to 98% of British Columbians and reached target speeds of 50 Mbps+ in 93% of households in the province, meaning that it is widely available for personal use [64]. Although these numbers are reduced in rural and remote communities, many of these same communities have strong local public services, such as libraries or community centers, which provide free access to internet and technology. Although not discussed by our rural participants, future focus groups with Indigenous and immigrant women will continue to assess the accessibility of eHealth options to ensure that they are a feasible option for all potential users.

Finally, although out of the scope of this analysis, which pooled all 7 focus groups from across BC into one assessment, the geographic location expectedly played a role in the needs of women experiencing PPD. When discussing a web-enabled intervention, women based in urban areas—especially the Lower Mainland of BC—expressed a need to connect with local in-person resources, whereas women from rural and remote communities, who do not have in-person resources available, required a support that could be used as a stand-alone, first-line resource. Research has shown that women in rural settings have lower rates of PPD detection and treatment, which leads to health disparities in these populations [65], further bolstering the importance of exploring the diverse needs of women experiencing PPD to create an accessible and acceptable intervention that fills the gap.

### Current eHealth Interventions for PPD

Patient engagement and acceptability are often overlooked in interventions using eHealth to treat perinatal mental health concerns. All existing interventions in this field rely solely on delivering psychotherapy via technology [24-30]. As evidenced by this study, this approach to management of PPD fails to address many of the key unmet needs of women. Some of these missed opportunities include providing education to address a lack of knowledge surrounding PPD, including peer support and evidence-based information, and incorporating partners in care to support women. These findings, along with recurring discussions of time constraints and lack of motivation that come with new parenthood and depression, indicate a need to shift away from providing a self-guided therapy-based intervention and to move toward a psychoeducational approach.

Psychoeducational eHealth interventions provide evidence-based information on a range of health topics and may be used for prevention, education, or treatment purposes [66]. A quick internet search will provide several psychoeducational eHealth resources for perinatal mental health; however, few of these resources have published information about their development or evaluation. The available literature highlights the low quality of current websites and apps for perinatal mental health, highlighting a serious need for evidence-based, patient-centered resources [67,68]. This has left an opportunity to design and develop an eHealth intervention that can truly meet the needs of women experiencing PPD, which this study will be used to inform.

### Next Steps

Looking toward web development, the design of this resource will be guided by the needs and preferences expressed by participants, with end user engagement at each phase of research to ensure that the patient’s voice is centered in all decision making. This will include a multidisciplinary advisory committee guiding web development, patient partners involved in discussions of privacy and security, and appropriately representative participant samples in each phase of future research. Our aim is to ensure that end users are able to provide feedback and guidance to co-design a resource that addresses their needs, although compromise may be necessary for the feasibility of some features, especially those with potential privacy and security threats. The next phase of this research will gather qualitative feedback from Indigenous and immigrant women to ensure that the developed web-enabled program is culturally safe and meets the needs of all Canadian women experiencing PPD. Following the integration of the new focus group data into the intervention content, usability testing will be undertaken before the intervention is tested in a randomized controlled trial to ensure its efficacy in reducing depressive symptomatology in women experiencing PPD. Future research should explore women’s experiences of PPD in low- and middle-income countries to determine how this digital health tool could be adapted to provide accessible and affordable care in these settings.

### Conclusions

This research provided a breadth and depth of insight into the needs of women experiencing PPD that has not been seen before in the literature, specifically as it relates to informing the development of an eHealth intervention. Participants described a multitude of unmet needs that persist because of varying gaps in care and knowledge; however, they also uncovered opportunities to bridge those gaps and meet their needs, in the interests of gaining ownership over their mental health journeys. These findings suggest that a web-enabled intervention is perceived as a welcome addition to supplement currently available resources for PPD and provide concrete recommendations on how it can best serve this purpose. Overall, this study provides an exciting foundation for creating an accessible, acceptable, and effective resource to assist in the treatment of PPD.

### Limitations

Despite these findings and the many strengths of the study protocol, gaps remain in the knowledge base that this study was unable to fill. First, based on the difficulty of recruiting for focus groups, we were unable to assess the needs of extremely rural and remote communities, where care options are already scarce. In addition, as this phase of the research included solely non-Indigenous, nonmigrant Canadian women, the sample does not reflect the values and preferences of Canada’s Indigenous and migrant populations. However, this study is part of a larger research project, which will assess the needs of Indigenous women and immigrant women who have experienced PPD, in the interests of representing and understanding the diverse needs of Canadian women.

In addition, the use of focus groups has some well-established limitations. As focus groups necessitate having multiple participants, ensuring recruitment and attendance can be difficult [69]. This study struggled with recruitment, resulting in some focus groups having only 2 attendees. In addition, a common phenomenon in focus group discussions is *group-think* or the conversation becoming biased because of dominant personalities and the need to fit in [69]. Further, some people tend to be overrepresented in the transcripts of focus group sessions, whereas others are silenced [69]. However, one of the main strategies to combat these limitations is to employ experienced, well-trained facilitators [69,70]. By including multiple facilitators in each focus group session—one who is extremely familiar with the literature and study and another who is familiar with the local community and available services for PPD—we were able to mitigate some of these potential outcomes. Despite these limitations, focus groups are thought to be extremely useful in projects that involve assessing the needs of a population or evaluating proposals because of the ease of quickly building off each other in discussions to form ideas. Moreover, for this particular population of women, who are often isolated and ashamed of their experiences, the opportunity to meet and speak with peers to share their stories was expressed as being very therapeutic.

## Appendix

Multimedia Appendix 1 Focus group interview guide.

## Abbreviations

BC: British Columbia
CBT: cognitive behavioral therapy
PPD: postpartum depression
QD: qualitative description
