# Supplementary material for: Digital Health Needs of Women With Postpartum Depression: Focus Group Study
Source: J Med Internet Res. 2021 Jan 6;23(1):e18934. doi: 10.2196/18934 (PMC7817361; doi:10.2196/18934)
Supplement: Multimedia Appendix 1 [file jmir_v23i1e18934_app1.docx]

**SUPPORT Focus Group Interview Guide**

*Introduction*

Hello, my name is **(name)** and I will be moderating today’s focus group session, alongside **(name of co-facilitator)**. This is **(name of note-taker)** and she/he/they will be assisting today, taking notes and handling the logistics of this session. I’d like to start by thanking each of you for taking the time to participate today. This session will run for approximately 2 hours, including the time for each of you to review the consent forms and study information, as well as to help yourself to the refreshments provided. If you would like anything to eat or drink, please feel free to help yourself now, so that the flow of our session is not disrupted once we get into our discussion.

*Purpose*

The purpose of today’s session is to gather your opinions and ideas regarding your experiences with postpartum depression (PPD) and its treatment options, as well as your feelings towards the use of technology in healthcare. We will be using the information and perspectives you provide to inform the creation of a web-enabled treatment intervention for women who are diagnosed with postpartum depression.

As mentioned, myself and **(name of co-facilitator)** will be leading our discussion today by asking questions, then encouraging and moderating the discussions that emerge. I would like to inform you all that this focus group session will be audio recorded to allow for analysis of the themes that emerge and to better inform our work. The identities of all participants will remain confidential in these recordings.

*Ground Rules*

Before we begin, there are some ground rules I would like to go over to ensure a respectful and free-flowing conversation:

1. Only one person may speak at a time. This is important for both the respect of everyone involved, as well as for the usefulness of our audio recording of the session. In this vein, please avoid any side conversations.
2. Please ensure that any cell phones are off or silenced, unless absolutely necessary. If you are required to take a phone call, please do so quickly and quietly away from the group.
3. You do not have to give a response for every question; however, I would like to hear from each of you at some point in our discussions and will try to make sure that everyone receives a chance to speak.
4. This discussion will be confidential in that I will not be using any of your names in any of the write-ups or data analysis that occurs in this project. However, I would like to stress to you all that, to keep these sessions confidential, we ask that you do not use names or anything directly identifying when discussing your personal experiences. We also ask that you do not discuss other participants’ responses outside of this session.
5. There are no “right” or “wrong” answers, just different opinions. We ask that you respect each others’ opinions and experiences, and say what is true for you without fear of judgement.
6. Let me know if you need a break. The bathrooms are **(location)**.
7. Your participation is completely voluntary, and you can leave at any time.
8. Finally, do whatever you need to do to take care of yourself (and your baby) over the next 2 hours, and let the facilitators know if you need anything to help with that.

Are there any questions before we move on?

*Participants Introductions*

Before we start, I’d like for all of us to get to know a little bit more about each other. Please tell me:

1. Your name
2. Your favourite place in BC and why?

*Questions*

1. Tell us whatever you feel comfortable sharing about your experience with PPD.
   1. Did you seek support or treatment for your PPD?
      1. If so, from who?
      2. If so, did it work?
         1. What specifically worked best?
      3. If so, for how long did you draw on those resources?
   2. How did you feel about the availability of resources for treatment/recovery?
      1. Did you feel as though the resources provided to you were culturally sensitive and safe for you?
      2. What are some of the online resources, if any, you’ve used to support your recovery?
2. How do you feel about using technology to support PPD treatment?
   1. Which types of technology do you think would be most beneficial if used this way?
3. Imagine that there is an app or website with scientifically proven skills to help you manage PPD. Would you use it?
   1. What would make you use it? What features would it have to have?
   2. What types of interactive components do you think would be most useful? (i.e. videos, quizzes, etc.)

***Show Bounce Back website features, including trailer video and example exercises (*https://cmha.bc.ca/programs-services/bounce-back/)*. Ask participants to point out what they do and don’t like about the platform, and what needs to be done differently to be acceptable to PPD.***

1. How would you feel about your partner being involved in your PPD treatment regimen?
2. What would you say was the most important factor in your recovery?
3. Of all the things we have discussed today, what would you say is the most important to take into account when we design this intervention?

*Closing*

**(Read back a summary of what has been discussed.)**

Do you feel that this summary accurately represents what has been discussed here today?

Does anyone have any final questions, comments, or concerns?

We have come to the end of our session today. I want to thank you all for your honest opinions and input on this topic. This is an extremely important stage of this project, and you were extremely helpful in providing useful feedback.

*If they come up organically:*

If you had access to discussion forums with other moms who have/had PPD, how often would you read posts or post yourself?

When using an online program to treat PPD, would you like to receive a customized series of workbooks?

When using an online program to treat PPD, would you make use of 4 to 6 coaching sessions delivered by phone or video conference? If so, who do you think should lead these coaching sessions?

When using an online program to treat PPD, how would you feel about having access to an optional reminder/notification system?
